# The Relationship between Anterior Chamber Angle and Intraocular Pressure Early after V4c Implantable Collamer Lens Implantation

**DOI:** 10.1155/2020/4014512

**Published:** 2020-07-21

**Authors:** Zongli Hu, Chunlin Chen, Min Sun, Rongdi Yuan, Jian Ye

**Affiliations:** ^1^Department of Ophthalmology, Daping Hospital, Army Medical University, Chongqing 400042, China; ^2^Department of Ophthalmology, Xinqiao Hospital, Army Medical University, Chongqing 400037, China

## Abstract

**Purpose:**

To confirm the relationship between anterior chamber angle (ACA) and intraocular pressure (IOP) early after V4c implantable collamer lens (ICL) implantation.

**Methods:**

Patients were assigned to two groups: (1) right eyes (control group) and (2) left eyes (experimental group), with miosis conducted immediately after ICL implantation in the left eyes. IOP, angle opening distance (AOD), trabecular-iris angle (TIA), and pupil diameter (PD) were compared between two groups at postoperative hours 1, 2, and 24. The relationship between ACA, PD, and IOP was analyzed by multiple linear regression.

**Result:**

Thirty-six eyes of 18 patients were enrolled. The prevalence of ocular hypertension (OHT, defined as IOP ≥ 21 mmHg) was 61.11% and 16.67% in the right and left eyes, respectively, (*χ*^2^ = 7.481, *p*=0.006). At postoperative hours 1 and 2, IOP and PD were significantly higher (*p* < 0.001) in the right eyes, and TIA and AOD were significantly lower (*p* < 0.05) in the right eyes than in the left eyes. There was no significant difference at 24 h postoperative in these parameters. After the right eye ICL implantation, the changes of AOD 500 and PD were both linearly correlated with postoperative IOP change (*β* = −23.707 and 1.731, respectively; *p* = 0.013 and 0.002, respectively).

**Conclusion:**

The ACA was significantly narrowed immediately after V4c ICL implantation. There was a negative linear correlation between ACA and early IOP and a positive linear correlation between PD and early IOP. We recommend the use of intracameral miotics immediately after V4c ICL implantation to reduce the incidence of IOP spikes.

## 1. Introduction

The Visian Implantable Collamer Lens (ICL) (STAAR Surgical Co., Monrovia, CA, USA) is widely applied to correct refractive errors in patients with moderate to high myopia. One of the postoperative complications is elevated intraocular pressure (IOP) [1, 2]. Although studies [3–7] of the current V4c ICL have shown that ocular hypertension (OHT) is rarely observed at 1 week, 1 month, 3 month, 6 month, 1 year, or 2 year postoperatively, few studies have focused on postoperative IOP in the first 24 h. Chen and Sri found that the prevalence of OHT was 31.82% [8] and 60% [9] at 24 h after V4c ICL implantation and that most OHT occurred within 1 h. OHT can lead to irreversible damage to the optic nerve, and the high rate of OHT in the early postoperative period is worthy of attention. Some studies [10, 11] reported that the ACA narrowed by 39–45% 1 month after surgery of V4c ICL implantation, and the mean IOP was higher than that before surgery. The aim of this study was to access the probable relationship between narrowed ACA and the high rate of OHT within 24 h after V4c ICL implantation and to determine if miosis was effective in control of early IOP.

## 2. Materials and Methods

### 2.1. Patients

This study was a prospective interventional case series. Institutional Review Board (IRB)/Ethics Committee approval was obtained prospectively by the Daping Hospital Institutional Review Board, Chongqing, China. The study was carried out from October 2017 to January 2018, and the setting was the Department of Ophthalmology, Daping Hospital, Army Medical University. The clinical trial registration number is ChiCTR-OON-17012838 (http://www.chictr.org.cn), and this study followed the tenets of the Declaration of Helsinki. All patients signed consent forms after receiving an explanation of the nature and possible consequences of the study.

The inclusion criterion was stable myopic error ranging from -3.00 D to -12.00 D for 12 months. The exclusion criterion was a history of ocular pathology, such as OHT, ocular trauma, retinal detachment, glaucoma, cataract, amblyopia, or ocular inflammation. Patients with anterior chamber depth (ACD) lower than 2.8 mm and patients over 45 years old were also excluded from this study.

### 2.2. Groups and Interventions

Patients implanted with V4c ICL were assigned to two groups: (1) right eyes, which served as the control group and received no miotic agent application after surgery; and (2) left eyes, which served as the experimental group and were submitted to miosis immediately after ICL implantation to narrow the pupil diameter (PD). IOP, angle opening distance (AOD), trabecular-iris angle (TIA), vault, and PD were compared between the right and left eyes at postoperative 1, 2, and 24 hours. The relationship between ACA and IOP was analyzed by multiple linear regression.

### 2.3. V4c ICL Implantation Surgical Procedure

All V4c ICL implantation surgeries were performed by the same ophthalmic surgeon. Compound tropicamide eye drops (1 ml: 5 mg: 5 mg, Santen, Japan) were applied 30 min before surgery to dilate the PD to 7 ± 0.5 mm. After the application of oxybuprocaine hydrochloride eye drops (20 ml: 80 mg, Santen, Japan) for topical anesthesia, a 3 mm temporal corneal incision and an auxiliary incision at 12 o'clock were made. The anterior chamber was injected with sodium hyaluronate (Shanghai Kinsson, China), a viscoelastic agent (OVD). The V4c ICL was inserted into the anterior chamber with a MicroSTAAR injector (STAAR Surgical Co., Monrovia, CA, USA) and then placed in the posterior chamber and positioned in the horizontal axis. OVD was exchanged with balanced salt solution. A 0.1 ml carbachol injection (2 ml: 0.1 mg, Freda, China), as the miotic agent, was instilled in the anterior chamber of the left eyes. After surgery, prednisolone acetate ophthalmic suspension (5 ml: 50 mg, Allergan, Ireland) and levofloxacin eye drops (5 ml: 24.4 mg, Santen) were administered topically four times daily for 2 weeks. No more mydriatic drops were applied between the procedure and each follow-up visit.

### 2.4. Outcome Measures

A noncontact tonometer (TX-20 full auto tonometer, Cannon, Japan) was used by a single skilled technician to detect IOP, and 3 parallel measurements were taken at each time point. OHT was defined as an IOP ≥ 21 mmHg.

**Figure 1:**
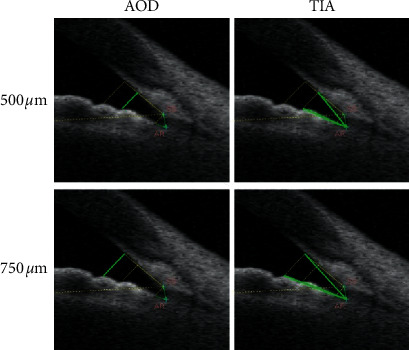
The indication of parameters measured by AS-OCT. Note: AOD: the vertical distance from the posterior surface of the cornea to the iris surface, with a 500 *µ*m or 750 *µ*m distance to the scleral spur; TIA: a vertical line was drawn from the cornea to the iris, and the TIA was the angle formed by the recess, cornea point, and iris point, with the recess as the vertex, and the cornea point was 500 *µ*m or 750 *µ*m from the scleral spur. Green line: the indication of a certain parameter. Abbreviations: AS-OCT: anterior segment optical coherence tomography, AOD: angle opening distance, and TIA: trabecular-iris angle.

The ICL in this study was positioned in the horizontal axis by the same ophthalmic surgeon, and we focused on a horizontal measurement of 0–180° in this study. Anterior segment optical coherence tomography (AS-OCT, CASIA SS-1000, Tomey, Japan) measurements were performed by a single technician, and both nasal and temporal data were recorded. The analyzed parameters included AOD 500, AOD 750, TIA 500, TIA 750, PD, and vault. The measurement and definition of these parameters are described as follows: (1) AOD: the vertical distance from the posterior surface of the cornea to the iris surface; (2) TIA: a vertical line drawn from the cornea to the iris and defined as the angle formed by the recess, cornea point, and iris point, with the recess as the vertex; (3) PD: the distance between the nasal and temporal iris; and (4) vault: the distance from the posterior surface of the intraocular lens (IOL) to the anterior surface of the crystalline lens. The definitions and representations of AOD and TIA are shown in Figure 1. These parameters were measured before surgery and at postoperative 1, 2, and 24 hours.

### 2.5. Statistical and Mathematical Analyses

Statistical analyses were performed with SPSS version 24.0 software (IBM, Armonk, NY, USA), and continuous variables are presented as the mean ± standard deviation (SD). Normality was checked by the Shapiro–Wilk test. Continuous variables accorded with normal distribution were analyzed by Student's *t*-test or one-way ANOVA, and comparisons were performed by the least significant difference (LSD) method. Comparison of data not accorded with normal distribution was analyzed by the Mann–Whitney *U* test. A chi square test was used to compare differences in qualitative data. Multiple linear regression was applied to analyze the relationship between ACA, PD, and IOP. The sample size for this study was based on an earlier study, and the required number of eyes per group was at least 10 with *α* = 0.05 and *β* = 0.1. A *p* value < 0.05 was considered statistically significant.

## 3. Results

A total of 18 patients (36 eyes) were enrolled in this study and included 8 males and 10 females with an average age of 21.78 ± 3.65 years. All surgeries were performed uneventfully with no significant complications. A miotic agent was applied in all left eyes at the end of surgery to achieve a PD of 3 ± 0.5 mm.

### 3.1. Fluctuations of IOP and ACA in the Right and Left Eyes after V4c ICL Implantation

Preoperative and postoperative (1, 2, and 24 hours) data of the right and left eyes are summarized in Table 1. The data of IOP, TIA 500, TIA 750, AOD 750, and vault in the right or left eyes at each time points were accorded with normal distribution (*p* > 0.05), and they were presented as mean ± SD. Data of AOD 500 and PD were not accorded with normal distribution, and they were presented as median (P25 and P75).

In the right eyes, mean IOP was significantly higher at postoperative 1 and 2 hours than before surgery (*p* < 0.001; ·*p*=0.004). Mean TIA 500, TIA 750, and AOD 750 and median AOD 500 were all significantly lower at postoperative hours 1, 2, and 24 than before surgery (*p* < 0.001). Mean TIA 500 and TIA 750 were higher at 24 h than at 1 h and 2 h postoperative; mean AOD 750 and median AOD 500 were higher at 24 h than at 1 h postoperative (*p* < 0.05). Median PD was significantly higher at postoperative hours 1 and 2 than before surgery (*p* < 0.001). Mean vault was lower at 2 h than at 1 h (*p*=0.002) and lower at 24 h than at 2 h (*p*=0.008).

In the left eyes, there were no significant differences in IOP before surgery and at postoperative hours 1, 2, and 24 (*p* > 0.05). Mean TIA 500, TIA 750, and AOD 750 and median AOD 500 were all significantly lower at postoperative hours 1, 2, and 24 than before surgery (*p* < 0.001), while there were no significant differences among the measurements obtained at postoperative time points (*p* > 0.05). Median PD was higher at 24 h than at 1 h and 2 h postoperative (*p*=0.003; ·*p*=0.001). Mean vault was higher at 24 h than at 1 h and 2 h postoperative (*p*=0.010; ·*p*=0.020).

### 3.2. Comparisons of Early Postoperative IOP and ACA in the Right and Left Eyes

At postoperative hours 1 and 2, IOP was significantly higher in the right eyes than in the left eyes (*p* < 0.001; ·*p* < 0.001), while there was no significant difference in IOP between the right and left eyes before or at 24 h after surgery (*p*=0.577; ·*p*=0.488). OHT was mostly observed at 1 h after V4c ICL implantation. The prevalence of postoperative OHT was 61.11% (11/18) and 16.67% (3/18) in the right and left eyes, respectively, and this difference was significant (*χ*^2^ = 7.481, *p*=0.006).

**Figure 2:**
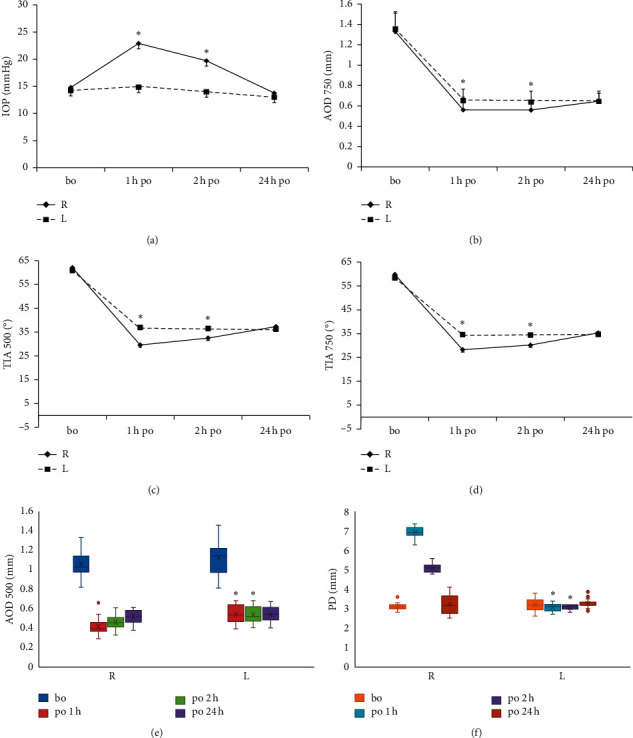
Comparison between the right and left eyes. Note: comparison in terms of (a) mean IOP, (b) mean AOD 750, (c) mean TIA 500, (d) mean TIA 750, (e) median AOD 500, and (f) median PD before surgery and at 1, 2, and 24 hours after surgery. ^*∗*^The difference was significant (*p* < 0.05) between the right and left eyes. Abbreviations: R: right eyes; L: left eyes; bo: before operation; po: postoperation; IOP: intraocular pressure; PD: pupil diameter; TIA: trabecular-iris angle; and AOD: angle opening distance.

Mean TIA 500, TIA 750, and AOD 750 and median AOD 500 were all significantly higher in the left eyes than in the right eyes at 1 h (*p* < 0.001; ·*p* < 0.001; ·*p* < 0.001; ·*p*=0.029) and 2 h (*p*=0.008; ·*p*=0.006; ·*p*=0.008; ·*p*=0.025) postoperatively, while there were no significant differences at 24 h after surgery (*p* > 0.05). At 1 and 2 hours postoperatively, median PD and mean vault were significantly higher in the right eyes than in the left eyes (*p* < 0.001), with no significant difference observed at postoperative hour 24 (*p* > 0.05). The comparisons of IOP and ACA before and after surgery are shown in Table 1 and Figure 2.

### 3.3. Relationship between ACA, PD, and Early Postoperative IOP after V4c ICL Implantation

**Figure 3:**
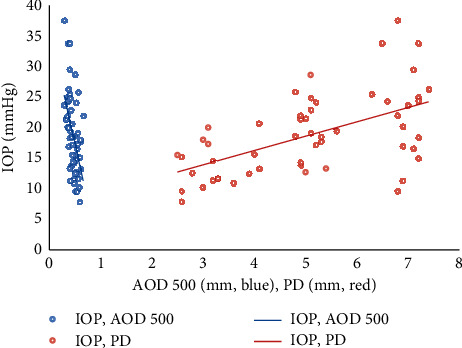
The relationship between anterior chamber angle (ACA), PD, and early postoperative intraocular pressure (IOP) after V4c ICL implantation. Note: the changes of AOD 500 and PD were both linearly correlated with postoperative IOP change (*β* = −23.707 and 1.731, respectively; *p*=0.013 and 0.002, respectively). Abbreviations: PD: pupil diameter; IOP: intraocular pressure; and AOD: angle opening distance.

ACA fluctuated in the right eyes as PD gradually decreased from approximately 7 mm to approximately 3 mm after ICL implantation. The data of dependent variable IOP were accorded with normal distribution (*p* > 0.05). In univariate linear regression, there were significantly negative correlations between TIA 500, TIA 750, AOD 500, AOD 750, and IOP (*p* ≤ 0.001) and a significantly positive correlation between PD and IOP (*p* < 0.001). After multiple linear regression (Figure 3), the two variables in AOD 500 and PD went into regression equation. After V4c ICL implantation, the changes of AOD500 and PD were both linearly correlated with postoperative IOP change (*β* = −23.707 and 1.731, respectively; *p*=0.013 and 0.002, respectively).

## 4. Discussion

There are several reasons that IOP increases after V4c ICL implantation. Residual viscoelastic agent and the glucocorticoid response [1, 12] are considered to be the leading causes of OHT within the first 24 h and at 2–4 week after surgery, respectively. We found that OHT was common (>50%) at 24 h after surgery even after adequate flushing of the viscoelastic agent. There is a lack of research on the relationship between anterior angle structures and early postoperative IOP within the first 24 h after V4c ICL implantation.

Ganesh S. [9] found that IOP generally peaked at 2 h after surgery, consistent with our results. In the study by Ganesh and colleagues, no miotic agent was used during or after surgery, and dorzolamide was topically applied to all patients to prevent OHT. In a study of early IOP after V4c ICL implantation, no acute OHT was observed in 3 h, 6 h, and 24 h postoperatively [13], but brinzolamide/timolol was applied twice daily to control IOP. In our study, although a miotic agent was applied at the end of surgery in the left eyes, no conventional IOP-lowering agent was applied after surgery. In other studies of V4c ICL [14–16], IOP was measured at 1 day and 1 week after surgery, and these studies showed that IOP was stable, at approximately 20 mmHg. Whereas in our study, OHT was usually observed within 1 h after surgery, and postimplantation OHT has rarely been reported in previous studies. The difference in results among these studies is likely related to the selection of postoperative observation time points.

While residual viscoelastic agent is considered the main cause of OHT after ICL implantation, we found that the prevalence of OHT was 61.11% in the right eyes, even though the OVD was adequately flushed by a single skilled surgeon in our study. Similarly, Chen and Sri [8, 9] found that the prevalence of OHT was 31.82%–60% at 24 h after V4c ICL implantation with no miotic agent application. We applied a noncontact tonometer to detect IOP to avoid contact measurement early after surgery in this study. Although the central corneal thickness (CCT) was important for IOP measurement by a noncontact tonometer, our aim was to confirm the relationship between change of ACA and IOP early after ICL implantation, and there was no change in CCT before or after surgery; so, the CCT was not considered a main factor to influence IOP. We speculate that in addition to OVD residue, the immediate changes in ACA that occur after ICL implantation might be the cause of increased IOP in the early postoperative period. In our study, ACA was significantly narrowed (TIA 500 decreased by 52.14% compared to preoperative) at 1 h after ICL implantation and slightly larger at 24 h than at 1 h after surgery (TIA 500 decreased by 39.87% compared to preoperative). IOP also recovered to a normal level at 24 h postoperative. In 2 prospective studies published in 2016 [17] and 2017 [11], ACA was measured 1 month after V4c ICL implantation and found to have been narrowed by 34–42% and 39–45%, respectively. In those studies, the ACA at 1 month postoperative was more open than the early ACA at 1 h postoperative in our study.

In this study, a significantly negative linear correlation was observed between AOD 500 and IOP, and a significantly positive linear correlation was observed between PD and IOP in 24 h after ICL implantation. These results indicate that the open degree of ACA did negatively affect early IOP. Similarly, after cataract surgery, the changes in AOD 500 was also found to be linearly correlated with postoperative IOP changes [18]. In another study published in 2016 [17], ACA was found to have been narrowed by 37% at 1 month after V4c ICL implantation, although no OHT was observed. However, ACA and IOP were not observed in the early postoperative period in that study. We speculate that the immediate changes that occur in ACA after ICL implantation might lead to OHT in the early postoperative period, and miosis might reduce the risk of OHT by opening the ACA.

The limitation of our study is that we measured IOP and anterior chamber parameters only within 24 h after operation. Whether these parameters remain stable over time in patients remain unknown. Thus, future studies with longer follow-up periods and larger populations are needed to determine the long-term relationships between ACA and IOP and whether miosis can effectively prevent OHT by opening the ACA.

## 5. Conclusions

The ACA was significantly narrowed immediately after V4c ICL implantation. There was a negative linear correlation between ACA and early IOP and a positive linear correlation between PD and early IOP. In order to reduce the incidence of IOP spikes, we recommend the use of intracameral miotics immediately after V4c ICL implantation.

## Figures and Tables

**Table 1:** Comparisons of early postoperative IOP and ACA after V4c ICL implantation in the right and left eyes.

		bo	1 h po	2 h po	24 h po
R	IOP (mmHg)	14.79 ± 2.79	22.91 ± 7.64	19.71 ± 4.53	13.76 ± 3.56
L		14.23 ± 3.11	14.85 ± 4.27 ^*∗∗∗*^	13.99 ± 3.63 ^*∗∗*^	13.00 ± 2.87
R	TIA 500 (°)	62.05 ± 5.20	29.59 ± 4.40	32.47 ± 3.88	37.31 ± 4.74
L		60.85 ± 5.75	36.95 ± 4.53 ^*∗∗∗*^	36.53 ± 4.78 ^*∗∗*^	36.27 ± 4.58
R	TIA 750 (°)	59.83 ± 4.68	28.28 ± 3.74	30.23 ± 3.52	35.32 ± 4.39
L		58.41 ± 4.46	34.63 ± 4.44 ^*∗∗∗*^	34.48 ± 5.08 ^*∗∗*^	34.77 ± 4.69
R	AOD 500 (mm)	1.03 (0.98, 1.14)	0.39 (0.38, 0.48)	0.45 (0.41, 0.51)	0.52 (0.46, 0.58)
L		1.14 (0.97, 1.22)	0.53 (0.46, 0.64) ^*∗∗∗*^	0.52 (0.47, 0.62) ^*∗∗*^	0.54 (0.48, 0.61)
R	AOD 750 (mm)	1.33 ± 0.18	0.56 ± 0.12	0.56 ± 0.09	0.64 ± 0.08
L		1.35 ± 0.17	0.65 ± 0.11 ^*∗*^	0.64 ± 0.11 ^*∗*^	0.64 ± 0.10
R	PD (mm)	3.10 (2.97, 3.20)	6.95 (6.80, 7.20)	5.10 (4.90, 5.22)	3.15 (2.75, 3.68)
L		3.20 (2.95, 3.42)	3.10 (2.90, 3.20) ^*∗∗∗*^	3.10 (2.98, 3.20) ^*∗∗∗*^	3.20 (3.18, 3.32)
R	Vault (*μ*m)	—	945.61 ± 187.82	746.61 ± 199.47	576.39 ± 167.66
L		—	406.94 ± 205.97 ^*∗∗∗*^	423.61 ± 200.57 ^*∗∗∗*^	580.44 ± 177.96

Note: data accorded with normal distribution are presented as mean ± SD, and data not accorded with normal distribution are presented as median (P25 and P75). The difference was significant between two groups at postoperative 1h and 2h (^*∗*^*p* < 0.05; ·^*∗∗*^*p* < 0.01; ·^*∗∗∗*^*p* < 0.001). Abbreviations: R: right eyes; L: left eyes, bo: before operation; po: postoperation, IOP: intraocular pressure, ACA: anterior chamber angle, TIA: trabecular-iris angle, AOD: angle opening distance, and PD: pupil diameter.

## Data Availability

The data used to support this study are available from the corresponding author upon request.
